# The development of visually guided stepping

**DOI:** 10.1007/s00221-019-05629-5

**Published:** 2019-08-30

**Authors:** Rachel Mowbray, Janna M. Gottwald, Manfei Zhao, Anthony P. Atkinson, Dorothy Cowie

**Affiliations:** 1 Department of Psychology, Durham University, South Road, Durham, DH1 3LE UK; 2 Department of Psychology, Uppsala University, Box 1225, 75121 Uppsala, Sweden

**Keywords:** Vision, Stepping, Reaching, Development, Posture

## Abstract

Adults use vision during stepping and walking to fine-tune foot placement. However, the developmental profile of visually guided stepping is unclear. We asked (1) whether children use online vision to fine-tune precise steps and (2) whether precision stepping develops as part of broader visuomotor development, alongside other fundamental motor skills like reaching. With 6-(*N* = 11), 7-(*N* = 11), 8-(*N* = 11)-year-olds and adults (*N* = 15), we manipulated visual input during steps and reaches. Using motion capture, we measured step and reach error, and postural stability. We expected (1) both steps and reaches would be visually guided (2) with similar developmental profiles (3) foot placement biases that promote stability, and (4) correlations between postural stability and step error. Children used vision to fine-tune both steps and reaches. At all ages, foot placement was biased (albeit not in the predicted directions). Contrary to our predictions, step error was not correlated with postural stability. By 8 years, children’s step and reach error were adult-like. Despite similar visual control mechanisms, stepping and reaching had different developmental profiles: step error reduced with age whilst reach error was lower and stable with age. We argue that the development of both visually guided and non-visually guided action is limb-specific.

## Introduction

For safe walking in complex environments, foot placement must be guided by visual cues about obstacles, depth, and ground texture. However, newborn infants are unable to make controlled, precise, visually guided steps. Independent standing and functional steps are not available for many months. However, by toddlerhood, children accumulate vast and varied walking experience (Adolph et al. 2012), and by 4 years, there is some evidence of adult-like visual behaviour during walking (Franchak and Adolph 2010). However, little research has directly tested how well children use this visual information to guide precise stepping. As a starting point, we look to the extensive literature on children’s visually guided reaching as a model of visually guided action.

### The development of precision stepping: insights from reaching

For reaching, there is a mid-childhood transition (Bard et al. 1990; Hay 1979; Hay et al. 1991; Pellizzer and Hauert 1996; Van Braeckel et al. 2007). Eight year old’s reaches are less accurate and slower than younger or older children’s (Bard et al. 1990; Hay et al. 1991; Pellizzer and Hauert 1996). Young children process visual and proprioceptive inputs relatively separately (Chicoine et al. 1992). In mid childhood, children begin integrating these inputs (Hay 1979; Van Braeckel et al. 2007). However, cortical regions associated with sensorimotor integration mature later than motor and sensory systems (Lenroot and Giedd 2006), causing a brief increase in reaching error.

Stepping might develop as part of broader sensorimotor development and, like reaching, show non-linear development. Adults’ steps and reaches have similar kinematic profiles and visual control mechanisms. They share a two-phase speed profile (Berthier and Keen 2006): first an acceleration phase brings the effector to the relevant area, then a deceleration phase for visually guided fine-tuning (Jakobson and Goodale 1991; Reynolds and Day 2005a; Zhao and Warren 2015). Adults rapidly update steps and reaches in response to visual change (Pisella et al. 2000; Reynolds and Day 2005b) and without vision, both steps and reaches are slower and less accurate (Babinsky et al. 2012; Berthier et al. 1996; Reynolds and Day 2005a; Smid and Den Otter 2013; Westwood et al. 2001).

Given the similar visual guidance of adults’ steps and reaches, we might also expect similarities in childhood. Starting with reaching, newborns make predictive arm movements to moving objects (von Hofsten 1980, 1982). Nine-month-olds’ reaches are kinematically different when vision is occluded (Babinsky et al. 2012). In mid childhood, reaching is less accurate without vision (Bard et al. 1990). For stepping, infant step frequency increases with visual stimulation (Pantall et al. 2011) and 3-year-olds rely on visual depth cues to control step descent (Cowie et al. 2010). Like reaching, stepping—for example in the context of obstacle crossing—remains immature in mid-childhood (Berard and Vallis 2006). But can children use online vision to fine-tune precise steps to a target? This visually guided precision is crucial for walking in natural environments (Chapman and Hollands 2007; Matthis et al. 2018).

The neural control of precise, visually guided action may be limb-general. The neural mechanisms of reaching may even have evolved from those controlling quadrupedal locomotion (Georgopoulos and Grillner 1989). Parietal regions control visually guided action in an effector-general manner (Tunik et al. 2007) and control the planning of upper (Buneo and Andersen 2006) and lower limb movement (Drew et al. 2008; Gwin et al. 2011). Precise stepping also engages prefrontal areas (Koenraadt et al. 2014) and is negatively affected by cognitive load (Alexander et al. 2005). However, we lack developmental evidence. Again, we look to reaching for clues: executive function correlates with reaching behaviour in infancy and childhood (Gottwald et al. 2016; Ruddock et al. 2016; Wilson and Hyde 2013). Given these ties between cognition and action and the protracted development of frontal regions (Blakemore and Choudhury 2006; Gogtay et al. 2004), we might predict that visuomotor development is overall long and supported by cognitive development.

### Stepping and reaching might have different developmental profiles

Despite the above-discussed similarities, developmental motor assessments commonly treat upper limb tasks, like grasping and reaching (fine motor) as qualitatively distinct from gross motor skills, like walking and balance (Cools et al. 2009). Further, the hands and feet are represented in distinct neural areas (Bracci et al. 2010; Dall’Orso et al. 2018). However, neural body representation tells us little about movement control. During adult movement, the neural coupling of the arms and legs is task dependent (Dietz 2002, 2018; Frigon 2017). For skilled, visually guided action, the arms are controlled by direct cortical-motoneuronal connections independently of the legs (Dietz 2003) but this does not necessitate asynchronous development of stepping and reaching.

Nonetheless, stepping and reaching do emerge at different times. Within months, infants can reach from a sitting posture (Thelen and Spencer 1998). Purposeful stepping, on the other hand, comes later. Infants must stand independently, before then learning to step in ways which promote stability (Moraes, Lewis, and Patla 2004; Roncesvalles et al. 2000) and to adjust active steps for careful foot placement. This poses a huge demand, given that balance remains immature long after walking onset (Brenière and Bril 1998; Godoi and Barela 2008; Woollacott and Shumway-Cook 1990). Nonetheless, just like stepping, reaching is crucially reliant on postural control. An infant must be able to stabilise the head and shoulders before they can reach successfully (Thelen and Spencer 1998). They must also develop anticipatory postural adjustments to support reaching (Witherington et al. 2002). In older children, postural stability correlates with manual dexterity (Flatters et al. 2014). Postural control is not a unique requirement of stepping—it underpins action more broadly.

In sum, evidence suggests that stepping and reaching are more similar than different. Both are visually guided in adulthood (Babinsky et al. 2012; Reynolds and Day 2005a), with similar kinematic profiles (Berthier and Keen 2006; Reynolds and Day 2005a), similar neural control mechanisms (Buneo and Andersen 2006; Drew et al. 2008; Tunik et al. 2007) and ties to cognition (Alexander et al. 2005; Gottwald et al. 2016) and postural stability (Flatters et al. 2014; Moraes et al. 2004). Further, both reaching (Bard et al. 1990; Hay et al. 1991; Pellizzer and Hauert 1996) and stepping (Berard and Vallis 2006) remain immature in mid childhood. Together, this evidence indicates that visually guided stepping and reaching might have similar developmental profiles.

### How might we measure stepping development?

To understand the development of precision stepping, we measured three different error types. Absolute error indicates the accuracy with which an individual can bring their effector to the target. Without vision, absolute error is increased for adult steps (Reynolds and Day 2005a). Variable error tells us how consistent steps are from one attempt to the next. Whilst variability tends to reduce with experience, it is an important feature of the learning process (Gliga 2018; Lee et al. 2017), allowing exploration of possibilities for action. Like absolute error, when vision is occluded, variability increases for adult steps (Reynolds and Day 2005a). Constant error (directional bias) might be particularly relevant for stepping. Adults preferentially step in ways that promote stability, widening or lengthening the base of support (Moraes et al. 2004). In other cases, constant error may represent maladaptive perceptual or response biases (Smid and Den Otter 2013). By considering multiple errors, we can address multiple hypotheses.

To our knowledge, this is the first study to map the development of visually guided precision stepping on flat ground. With children and adults, we manipulated visual input during steps and reaches in two directions. We hypothesised first that both steps and reaches would be visually guided (H1), with greater absolute and variable error with vision occluded (Chicoine et al. 1992; Cowie et al. 2010). Second, we hypothesised that stepping develops as part of broader visuomotor development, sharing a developmental profile with reaching (H2) with increased absolute and variable error in mid childhood (Bard et al. 1990; Hay et al. 1991; Pellizzer and Hauert 1996; Van Braeckel et al. 2007). Third, we hypothesised that step error would be affected by step direction (H3), with greater error for side steps, especially without vision (Reynolds and Day 2005a). We also expected side steps to be widened and straight steps lengthened, widening the base of support (Moraes et al. 2004). Finally, regarding postural stability (H4), we hypothesised that stability would correlate with step error, improve with age, and be poorer without vision (Woollacott and Shumway-Cook 1990).

## Methods

### Participants

All participants gave informed consent and had typical cognitive, motor and physical development, normal or corrected to normal vision, and right hand and foot dominance. For handedness and footedness participants/parents were asked which hand they/their child write(s) with and which foot they/their child normally kicks a ball with. We verified binocular depth perception in all participants with the Frisby stereo test (Frisby 1980).

Six-year-olds (*N* = 11, 5 female) had a mean age of 5.9 years (SD 0.2 years), mean leg length of 58.6 cm (SD 2.9 cm) and mean arm length of 49.6 cm (SD 2.7 cm). Seven-year-olds (*N* = 11, 3 female) had a mean age of 6.9 years (SD 0.1 years), mean leg length of 61 cm (SD 4.32 cm) and mean arm length of 52.8 cm (SD 2.4 cm). Eight-year-olds (*N* = 11, 3 female) had a mean age of 7.9 years (SD 0.4 years), mean leg length of 68.1 cm (SD 4.2 cm) and mean arm length of 55.6 cm (SD 2.6 cm). Adults (*N* = 15, 10 females) had a mean age of 25.9 years (SD 3.4 years) and mean leg length of 88.3 cm (SD 6.0 cm).

### Design and equipment

Children completed the reaching task and the stepping task (order counter-balanced). Adults completed the stepping task only. The development of visually guided stepping has been less extensively researched than the visual control of reaching, making an adult comparison group important for interpreting children’s step error. Both reaching and stepping tasks used a mixed design with two within-subjects variables: vision (on/off) and direction (ahead/side) and one between-subjects variable: age (6/7/8 years/adult). These age groups would allow us to identify an increase in error between 6 and 8 years (Bard et al. 1990; Hay et al. 1991).

We used Vicon motion-capture (240 Hz) with reflective markers on participants’ bare right foot on the second metatarsal head, front ankle, lateral malleolus and heel. For reaching, there was a single marker on the right index fingernail. To measure postural control, one marker was placed on each shoulder. Participants wore PLATO glasses throughout, allowing visual occlusion via a button press. We chose reach and step distances via piloting in which participants made a self-determined comfortable step/reach. Steps of 45% leg length, and reaches of 30% arm length, were consistently deemed comfortable.

For stepping, we marked start positions by tracing around the feet. We made step targets by cutting out a card trace of the participant’s right foot. We measured participant’s leg length from anterior superior iliac spine (pelvis) to medial malleolus (inner ankle). Required step length was scaled to leg length by sorting leg length into bands (band 1 > 30 cm ≤ 49 cm, band 2 ≥ 50 cm ≤ 69 cm, band 3 ≥ 70 ≤ 89, band 4 ≥ 90 cm < 109 cm) and scaling according to the average length for that band. We secured targets to the floor with Velcro: one target at 45% leg length straight ahead of the right foot start position, the second target at 45° to the right, also at a distance of 45% leg length. For example, a leg length of 62 cm falls into band 2, for which average leg length is 60 cm required step distance would be 18 cm (45% of 60 cm).

For reaching, a start position for the right index finger was marked by a star sticker on the table top. Star targets (diameter = 13 mm) were also placed on the table top. We measured participants’ arm length from shoulder to the end of the middle finger and scaled required reach length by sorting into bands (band 1 > 40 cm ≤ 49 cm, band 2 ≥ 50 cm ≤ 59 cm, band 3 ≥ 60 cm ≤ 69 cm, band 4 ≥ 70 cm < 80 cm) as per leg length. We placed one target at 30% arm length, straight ahead of the right finger start position. The second target was placed at 45° to the right, also at a distance of 30% arm length.

### Procedures

To measure postural stability, participants stood with feet shoulder width apart and were instructed to stand as still as possible for 30 s, then again with vision occluded. For the main task, participants made reaches/steps to targets with and without vision. For stepping, participants began standing on the start positions. For reaching, participants were seated with their right index finger on the start position. On each trial, the experimenter covered one of the targets (ahead or side) using card which was colour-matched to the surface.

For both steps and reaches, we asked participants to move in time with an audio track. This was four rhythmic tones, followed by the vocal: “drip, drop, splash!” (tones/words M 655 ms apart). Participants were required to begin their step or  reach on “drop”, land it onto the target on “splash” and then return to the start position. For stepping, we instructed participants to match their own foot exactly to the target foot. For reaching, we instructed participants to point to the middle of the target. The audio track was played on loop with a 7-s delay between trials, during which the experimenter set up the next trial by covering one of the targets. We instructed participants to look at the visible target ready for the next trial. In the vision off condition, we occluded vision on the word “drop”, which coincided with movement onset until the step/reach was complete. The only difference between the visual conditions was the availability of vision during the movement. Participants completed four blocks of ten trials for both steps and reaches in which conditions were randomised, with short breaks as needed.

### Analysis

**Fig. 1:**
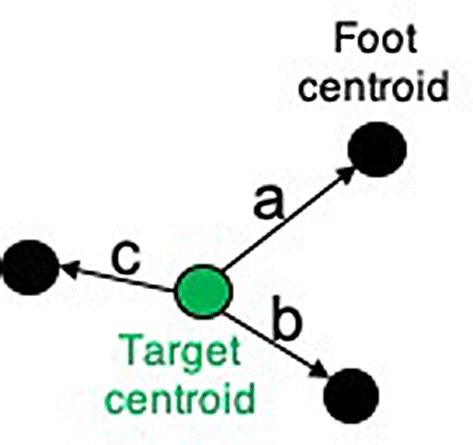
Target shown in green. Foot centroids shown in black. For step and reach error we calculated three error types—Constant error: e.g. signed mean value of distances a, b and c. Absolute error: e.g. unsigned mean value of absolute distances |a|, |b| and |c|. Variable error: e.g. standard deviation of absolute distances |a|, |b| and |c|

We recorded the locations of the start position and targets using motion capture. We filtered motion capture data using a 6-Hz low-pass Butterworth filter. A custom-written MATLAB script calculated the centroids of the start position and targets. The centroid (or centre of mass) of a shape is the mean position of all coordinates in the shape. We calculated error using the distance between the target centroid and foot/finger centroid at the end of the step/reach (Fig. 1). To analyse postural stability, we calculated the path length of the shoulder markers. This was the mean distance moved by the shoulder markers. We analysed the dependent variables using mixed model ANOVAs and Bonferroni-corrected post hoc tests. We calculated Partial correlations between shoulder path length and absolute error for both steps and reaches, controlling for age. Due to kurtosis in the data, we transformed the stepping, reaching and postural data by taking the square root of the values before calculating correlations.

We excluded trials where the participant did not do the task as instructed (e.g. used the left foot) or because of equipment error (e.g. PLATO glasses batteries were flat). For stepping, no adults had trials excluded. Three 6-year-olds had one trial excluded and one 6-year-old had three trials excluded. One 7-year-old had one trial excluded, one had two trials excluded and one had seven trials excluded. One 7-year-old was excluded from the stepping and reaching analysis entirely since they had 11 trials excluded (> 25% stepping data). One 8-year-old had one trial excluded. For reaching, one 6-year-old had two trials excluded and one had three trials excluded. Two 7-year-olds had one trial excluded and one 7-year-old had five trials excluded. One 8-year-old had one trial excluded.

## Results

For stepping, shoulder path length and reaching, we report main effects of vision, age and direction on: absolute, variable and constant error. There was only one significant interaction between vision and direction for variable step error. We also present correlations between step error, reach error and shoulder path length. We reiterate our hypotheses: H1—steps and reaches will be visually guided, with higher absolute and variable error when vision occluded; H2—stepping and reaching will share a developmental profile, with a mid-childhood peak in absolute and variable error; H3—step error will be affected by step direction, with higher error for side steps with vision occluded and a bias to widen the base of support; H4—step error will correlate with postural stability.

**Fig. 2:**
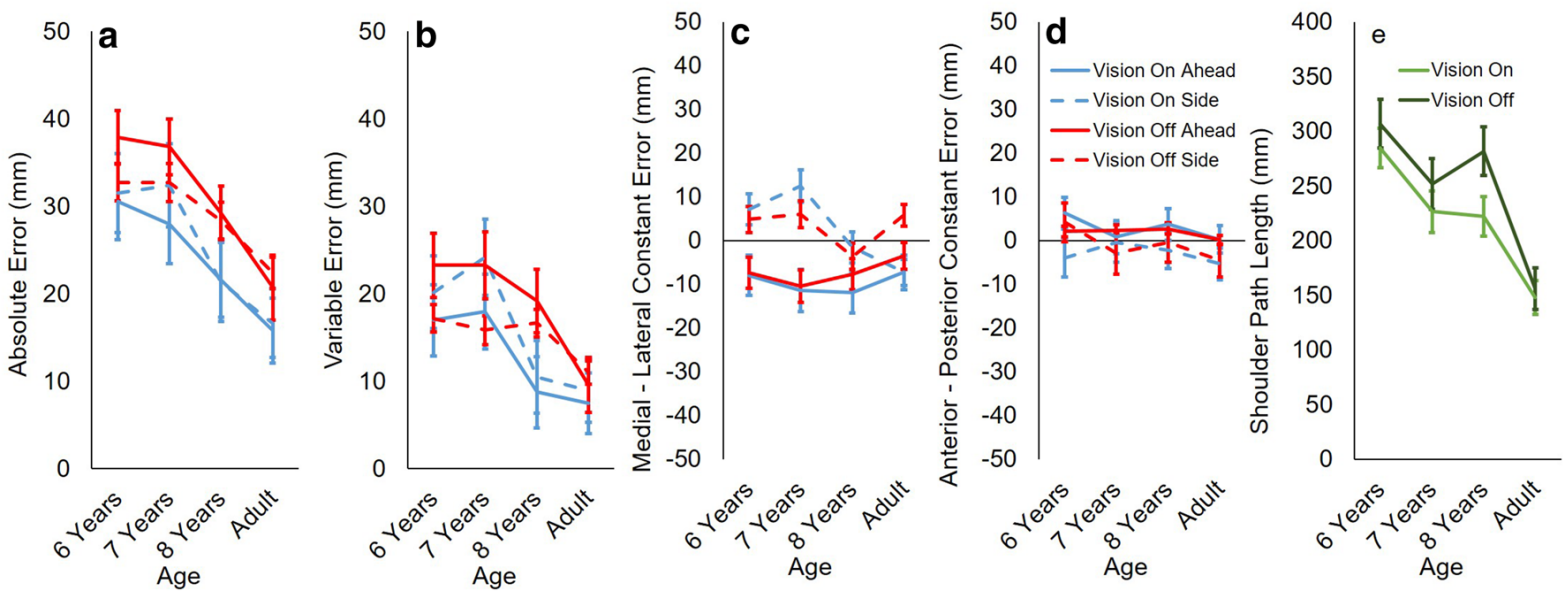
Step error and postural stability. Group means and standard errors for **a** absolute error; **b** variable error; **c** constant medial-lateral error; **d** constant anterior–posterior error; and **e** shoulder path length. Values are shown for both vision conditions (on/off) and both directions (ahead/side) at all ages. For medial-lateral error (**c**): negative values indicate left bias, positive values indicate right bias. For anterior–posterior error (**d**): negative values indicate backward bias, positive values indicate forward bias

In support of H1, absolute step error was significantly higher with vision occluded (M 30.1 mm, SE 1.0 mm) than with vision available (M 24.7 mm, SE 2.0 mm) *F*(1, 43) = 7.125, *p* = .011, $\eta_{\text{p}}^{2}$ = 0.142 (Fig. 2a). There were significant effects of age on absolute step error *F*(3, 43) = 8.079, *p* < .001, $\eta_{\text{p}}^{2}$ = 0.36 (Fig. 2a). However, contrary to H2, we did not find any increase in error in mid-childhood. Rather, children’s absolute step error was higher than adults’ (M 18.9 mm, SE 2.2 mm) at 6 years (M 33.2 mm, SE 2.5 mm, *p* = .001) and 7 years (M 32.5 mm, SE 2.7 mm, *p* = .002) with no significant difference in absolute step error between 6 and 7 years (*p* = 1.00). By 8 years (M 25.2 mm, SE 2.5 mm), absolute step error was adult-like (*p* = .413). The reduction in absolute step error between 7 and 8 years was not significant (*p* = .326). This effect of age cannot be explained by better task learning among older children and adults: we found no overall change in error between the first and last five trials (*p* = .141) and no interaction with age (*p* = .364). Contrary to H3, there was no effect of direction on absolute step error (*p* = .793).

Our results for variable step error partially support H1. Whilst there was no main effect of vision on variable step error (*p* = .099), there was a significant interaction between vision and direction *F*(1, 43) = 8.559, *p* = .005, $\eta_{\text{p}}^{2}$ = 0.116 (Fig. 2b). For steps straight ahead, variable error was higher with vision occluded (M 30.8 mm, SE 1.8 mm) than with vision available (M 23.2 mm, SE 2.2 mm) *t*(46) = − 3.547, *p* = .001. For side steps, there was no effect of vision (*p* = .099). Therefore, H1 is largely supported, but qualified by step direction. There was a significant effect of age on variable step error *F*(3, 43) = 4.813, *p* = .006, $\eta_{\text{p}}^{2}$ = 0.251 (Fig. 2b). However, contrary to H2, post hoc tests did not reveal any significant differences between any of the child age groups for variable error (*p*’s > .3): 6 years (M 19.4 mm, SE 2.6 mm), 7 years (M 20.9 mm, SE 2.7 mm), 8 years (M 13.8 mm, SE 2.6 mm). However, variable error was generally higher in children, and significantly higher for 6-year-olds than adults (*p* = .029). There was no effect of direction on variable step error (*p* = .593).

There was no effect of vision or age on constant step error (*p*’s > .5). In support of H3, there was a significant effect of direction on medial–lateral constant step error *F*(1, 43) = 26.447, *p* < .001, $\eta_{\text{p}}^{2}$ = 0.381 (Fig. 2c) and on anterior–posterior constant step error *F*(1, 43) = 9.230, *p* = .004, $\eta_{\text{p}}^{2}$ = 0.177 (Fig. 2d). Participants had a medial bias in the ahead condition (M − 8.4 mm, SE 1.7 mm) and a lateral bias in the side condition (M 3.9 mm, SE 1.7 mm). Steps were biased forwards in the ahead condition (M 2.3 mm, SE 1.0 mm) and backwards in the side condition (M − 1.9 mm, SE 1.8 mm).

As predicted (H4), shoulder path length was significantly greater with vision occluded (M 249.2 mm, SE 10.69 mm) than with vision available (M 220.4 mm, SE 8.9 mm) *F*(1, 43) = 12.160, *p* = .001, $\eta_{\text{p}}^{2}$ = 0.220 (Fig. 2e). Also confirming H4, there was a significant effect of age on shoulder path length *F*(3, 43) = 12.923, *p* < .001, $\eta_{\text{p}}^{2}$ = 0.474 (Fig. 2e). Children of all ages (6 years—M 296.0 mm, SE 18.2 mm, *p* = < .001; 7 years—M 239.2 mm, SE 19,3 mm, *p* = .007; 8 years—M 252.0 mm, SE 18.2 mm, *p* = .001) had greater shoulder path length than adults (M 152. 2 mm, SE 15.6 mm). Contrary to H4, shoulder path length did not correlate with step error in any condition (*p*’s > .09).

**Fig. 3:**
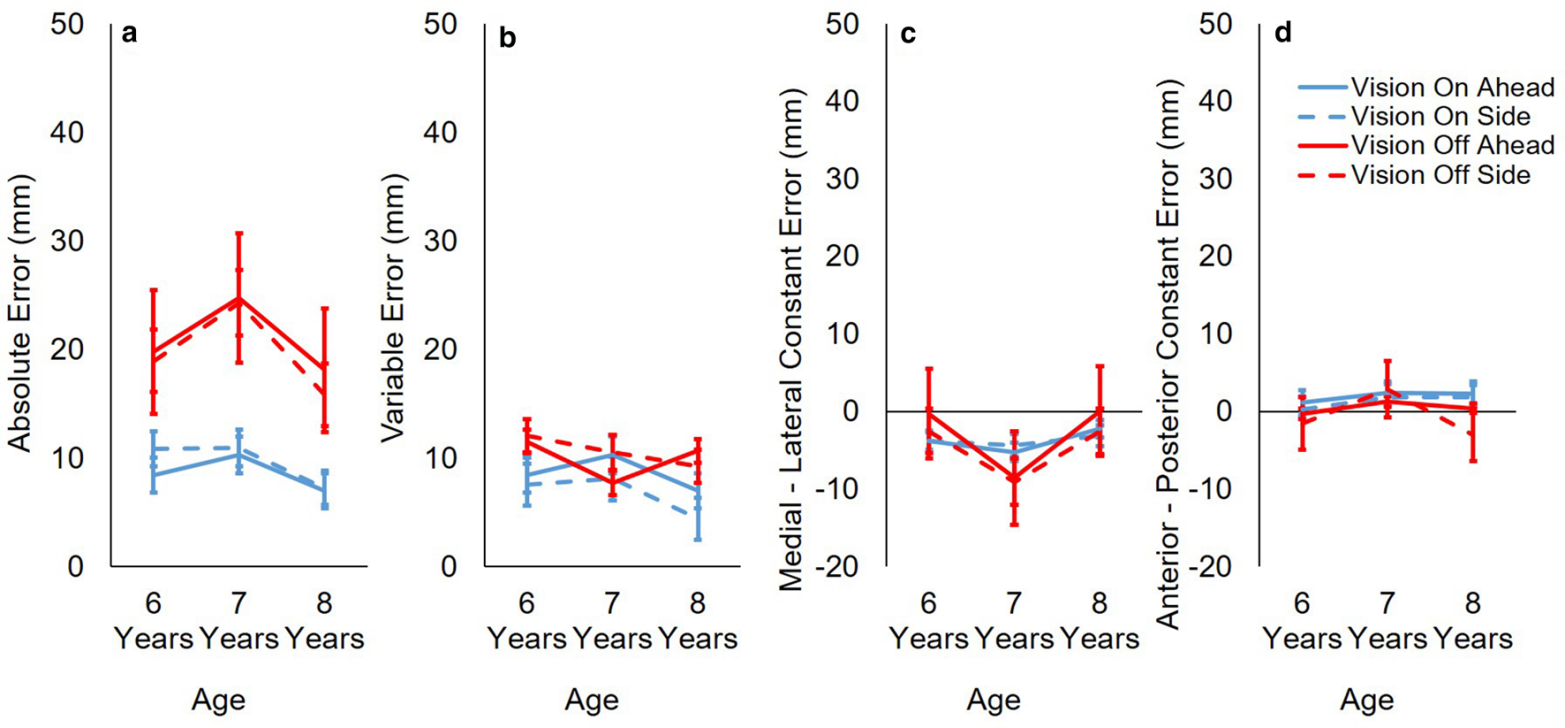
Reach error. Group means and standard errors for **a** absolute error; **b** variable error; **c** constant medial-lateral error; and **d** constant anterior–posterior error. Values are shown for both vision conditions (on/off) and both directions (ahead/side) at all ages (6/7/8 years). For medial-lateral error (**c**): negative values indicate left bias, positive values indicate right bias. For anterior–posterior error (**d**): negative values indicate backward bias, positive values indicate forward bias

In support of H1, absolute reach error was significantly greater with vision occluded (M 20.3 mm, SE 2.4 mm) than with vision available (M 9.1 mm, SE 0.9 mm) *F*(1, 29) = 34.375, *p* < .001, $\eta_{\text{p}}^{2}$ = 0.542 (Fig. 3a). Our predictions about age were not supported (H2). There was no effect of age or direction (*p*’s > .3) on absolute reach error. In support of H1, variable reach error was significantly greater with vision occluded (M 10.29 mm, SE 0.65 mm) than with vision available (M 7.6 mm, SE 0.9 mm) *F*(1, 29) = 9.115, *p* = .005, $\eta_{\text{p}}^{2}$ = 0.239 (Fig. 3b). However, contrary to H2, there was no effect of age on variable error (*p* = .359). There was no effect of direction on variable reach error (*p* = *.559*).There was no effect of vision, age or direction on constant reach error (*p*’s > .06). Shoulder path length did not correlate with absolute reach error in any conditions (*p*’s > .5).

We used a compromise power analysis in G*Power to assess the power of our analyses. We calculated implied power for detecting a large effect size (*F* = 0.3), with an alpha level 0.05, and beta/alpha ratio = 1. For stepping (children and adults), with a correlation among repeated measures of *r* = 0.47 (calculated from our data), our sample of *N* = 47 implies a power of 0.80 for between-subjects effects, 0.99 for within-subjects effects and 0.97 for interactions. For reaching (children only), with a correlation among repeated measures of *r* = 0.70 (calculated from our data), our sample of *N* = 32 implies a power of 0.72 for between-subjects effects, 0.995 for within-subjects effects and 0.99 for interactions.

## Discussion

Adults rely on vision to guide steps, especially when walking in complex, natural environments (Matthis et al. 2018; Reynolds and Day 2005a; Smid and Den Otter 2013). Nonetheless, little research has addressed visually guided stepping developmentally. We show that children’s precision stepping is visually guided (H1). However, unexpectedly (H2), we found the development of stepping was very different to reaching. Further, neither stepping nor reaching followed the non-linear developmental profile previously reported for reaching (Bard et al. 1990; Hay et al. 1991; Pellizzer and Hauert 1996; Van Braeckel et al. 2007). We now elaborate on these findings as well as on the directional biases in step placement (H3) and the relationship between step error and postural stability (H4).

### Children show adult-like reliance on vision for precision stepping

Children use online vision to control reaching (e.g. Bard et al. 1990; Chicoine et al. 1992). We show that children’s precision stepping is also visually guided. Most interesting of all, we found that children aged 6, 7 and 8 years rely on vision for stepping to the same extent as adults. At 6 and 7 years, children’s stepping error was overall higher than that for adults. However, the impact of visual occlusion on stepping error was equal at all ages. This suggests that children weight reliance on vision in an adult-like way. As hypothesised (H1), both steps and reaches were more accurate with vision available. Further, both reaches and steps straight ahead were more precise with vision available. We show that, like adults (Reynolds and Day 2005a; Smid and Den Otter 2013; Westwood et al. 2001), young children use online vision to fine-tune arm and leg movements and that stepping and reaching share similar visual control mechanism, likely controlled by parietal regions (Buneo and Andersen 2006; Drew et al. 2008; Gwin et al. 2011).

Also, in support of our first hypothesis (H1), steps were more variable with vision occluded. Interestingly, this is qualified by an interaction with direction, such that it occurs only for straight-ahead steps. In fact, we had anticipated (H3) that side steps would be more challenging, since they deviate from the normal forward movement trajectory of walking. However, the higher error for straight steps may reflect their narrower, less stable base, which is more easily compromised when vision is removed.

Previous work has shown that children use vision during step descent (Cowie et al. 2010), when walking in cluttered environments (Franchak and Adolph 2010) and when approaching obstacles (Berard and Vallis 2006). These complex and naturalistic tasks provide rich, ecological data. However, they do not show whether children use online vision to fine-tune active steps—especially when the landing location is very small (a single target). In this study, we have shown that children do use online vision to carefully guide the foot to a constrained landing location. This behaviour is crucial when walking in complex environments, where only certain, small footholds afford stable forward progression.

### Precision stepping and reaching have different developmental profiles

Based on the extensive literature on reaching (Bard et al. 1990; Hay et al. 1991; Pellizzer and Hauert 1996; Van Braeckel et al. 2007), we anticipated a non-linear developmental profile for stepping (H2). In contrast, stepping error decreased gradually and linearly with age. By 8 years, both accuracy and variability for stepping were adult like. This complements research showing adult-like step accuracy at 9 years during walking (Corporaal et al. 2018). Importantly, stepping error decreased with age both with and without vision. This suggests that development might be driven by improvements in proprioception, rather than by improvement in visual control.

In contrast, reaching error was stable between 6 and 8 years both with and without vision. We, therefore, show different developmental profiles for reaching and stepping and argue that both visually guided and non-visually guided actions develop in a limb-specific manner. This supports independent assessment of upper (fine) and lower limb (gross) movement in developmental motor assessments (Cools et al. 2009). We should expect upper and lower limb visuomotor control to typically develop at different rates. Stepping continues maturing long after reaching—just like controlled stepping emerges later than reaching in infancy (Berger and Adolph 2007). The neural control of precise movement of the arms and legs may be decoupled and develop asynchronously (Dietz 2003).

We found no change in reaching error between 6 and 8 years. This contrasts with other studies. Numerous studies show a non-linear developmental trend (Bard et al. 1990; Hay 1979; Hay et al. 1991; Pellizzer and Hauert 1996; Van Braeckel et al. 2007). However, in previous work, reaches were much larger (Bard et al. 1990; Hay 1979; Hay et al. 1991; Van Braeckel et al. 2007). In our task, children performed small reaches equally proficiently from 6 to 8 years, with reach error that was lower than (1) step error and (2) reach error in previous studies (Bard et al 1990). We argue that for our small reaches, children’s performance was mature.

### Does postural stability constrain precision stepping performance?

We predicted that biases in foot placement would widen and lengthen steps to increase stability (H3). However, our results only partially supported this. Steps were biased laterally (to the right) in the side condition. This bias widens the base of support. However, steps were also biased posteriorly in the side condition and medially in the ahead condition. Both of these biases narrow the base of support, arguably reducing stability. It is, therefore, possible that these biases are unrelated to stability and may be due to sensory or perceptual error.

Precision stepping requires children to guide the foot to a precise landing location, all whilst balancing on one leg. Since balance continues developing into adolescence (Godoi and Barela 2008), we expected that balance would constrain children’s stepping performance (H4). However, controlling for age, we found no correlation between postural stability and step error. We, therefore, argue that other factors—neural and cognitive development (Corporaal et al. 2017, 2018; Gogtay et al. 2004; Zelazo 1983), motor imagery (Sooley et al. 2018), internal models (Contreras-Vidal et al. 2005) and proprioception (King et al. 2010)—contribute to stepping development. Despite improvements in both postural stability and step error between 6 and 8 years, balance does not seem to be the most crucial factor in this simple, stepping task.

### Protracted and limb-specific development for visually guided stepping

Children use online vision to fine-tune precise steps. We, therefore, show that precision stepping shares a visual control mechanism with other motor tasks, like reaching. However, precision stepping takes longer to mature. We argue that the earlier emergence of reaching relative to stepping provides earlier, more extensive opportunity for children to practice reaching. This leaves stepping (both visually guided and non-visually guided) maturing relatively later than reaching.
